# Robotic-assisted management of Zinner syndrome: a case report and review of the surgical approach

**DOI:** 10.1093/jscr/rjaf162

**Published:** 2025-07-17

**Authors:** Bader Alsaikhan, Ahmed Alasker, Ahmed Nazer, Mohammad Alkhamees, Mohammad Alghafees, Abdulrahman A Bin Moammar, Turki Alferayan, Mohammed Alrashed, Abdulaziz Abdullah Alohali, Abdulaziz Almanie

**Affiliations:** College of Medicine, King Saud bin Abdulaziz University for Health Sciences (KSAU-HS), Sheikh Jaber Al-Sabah Road, Khashm Al An District, PO Box 3660, Riyadh 11481, Saudi Arabia; Division of Urology, Department of Surgery, King Abdulaziz Medical City, Ministry of National Guard Health Affairs, King Saud Bin Abdulaziz University for Health Sciences, Ar Rimayah District, PO Box 22490, Riyadh 11426, Saudi Arabia; King Abdullah International Medical Research Centre (KAIMRC), Ministry of National Guard Health Affairs, King Saud Bin Abdulaziz University for Health Sciences, Ar Rimayah District, PO Box 22490, Riyadh 11426, Saudi Arabia; College of Medicine, King Saud bin Abdulaziz University for Health Sciences (KSAU-HS), Sheikh Jaber Al-Sabah Road, Khashm Al An District, PO Box 3660, Riyadh 11481, Saudi Arabia; Division of Urology, Department of Surgery, King Abdulaziz Medical City, Ministry of National Guard Health Affairs, King Saud Bin Abdulaziz University for Health Sciences, Ar Rimayah District, PO Box 22490, Riyadh 11426, Saudi Arabia; King Abdullah International Medical Research Centre (KAIMRC), Ministry of National Guard Health Affairs, King Saud Bin Abdulaziz University for Health Sciences, Ar Rimayah District, PO Box 22490, Riyadh 11426, Saudi Arabia; College of Medicine, King Saud bin Abdulaziz University for Health Sciences (KSAU-HS), Sheikh Jaber Al-Sabah Road, Khashm Al An District, PO Box 3660, Riyadh 11481, Saudi Arabia; Division of Urology, Department of Surgery, King Abdulaziz Medical City, Ministry of National Guard Health Affairs, King Saud Bin Abdulaziz University for Health Sciences, Ar Rimayah District, PO Box 22490, Riyadh 11426, Saudi Arabia; King Abdullah International Medical Research Centre (KAIMRC), Ministry of National Guard Health Affairs, King Saud Bin Abdulaziz University for Health Sciences, Ar Rimayah District, PO Box 22490, Riyadh 11426, Saudi Arabia; College of Medicine, King Saud bin Abdulaziz University for Health Sciences (KSAU-HS), Sheikh Jaber Al-Sabah Road, Khashm Al An District, PO Box 3660, Riyadh 11481, Saudi Arabia; Division of Urology, Department of Surgery, King Abdulaziz Medical City, Ministry of National Guard Health Affairs, King Saud Bin Abdulaziz University for Health Sciences, Ar Rimayah District, PO Box 22490, Riyadh 11426, Saudi Arabia; King Abdullah International Medical Research Centre (KAIMRC), Ministry of National Guard Health Affairs, King Saud Bin Abdulaziz University for Health Sciences, Ar Rimayah District, PO Box 22490, Riyadh 11426, Saudi Arabia; College of Medicine, King Saud bin Abdulaziz University for Health Sciences (KSAU-HS), Sheikh Jaber Al-Sabah Road, Khashm Al An District, PO Box 3660, Riyadh 11481, Saudi Arabia; Division of Urology, Department of Surgery, King Abdulaziz Medical City, Ministry of National Guard Health Affairs, King Saud Bin Abdulaziz University for Health Sciences, Ar Rimayah District, PO Box 22490, Riyadh 11426, Saudi Arabia; King Abdullah International Medical Research Centre (KAIMRC), Ministry of National Guard Health Affairs, King Saud Bin Abdulaziz University for Health Sciences, Ar Rimayah District, PO Box 22490, Riyadh 11426, Saudi Arabia; College of Medicine, King Saud bin Abdulaziz University for Health Sciences (KSAU-HS), Sheikh Jaber Al-Sabah Road, Khashm Al An District, PO Box 3660, Riyadh 11481, Saudi Arabia; College of Medicine, King Saud bin Abdulaziz University for Health Sciences (KSAU-HS), Sheikh Jaber Al-Sabah Road, Khashm Al An District, PO Box 3660, Riyadh 11481, Saudi Arabia; Division of Urology, Department of Surgery, King Abdulaziz Medical City, Ministry of National Guard Health Affairs, King Saud Bin Abdulaziz University for Health Sciences, Ar Rimayah District, PO Box 22490, Riyadh 11426, Saudi Arabia; King Abdullah International Medical Research Centre (KAIMRC), Ministry of National Guard Health Affairs, King Saud Bin Abdulaziz University for Health Sciences, Ar Rimayah District, PO Box 22490, Riyadh 11426, Saudi Arabia; College of Medicine, King Saud bin Abdulaziz University for Health Sciences (KSAU-HS), Sheikh Jaber Al-Sabah Road, Khashm Al An District, PO Box 3660, Riyadh 11481, Saudi Arabia; Division of Urology, Department of Surgery, King Abdulaziz Medical City, Ministry of National Guard Health Affairs, King Saud Bin Abdulaziz University for Health Sciences, Ar Rimayah District, PO Box 22490, Riyadh 11426, Saudi Arabia; King Abdullah International Medical Research Centre (KAIMRC), Ministry of National Guard Health Affairs, King Saud Bin Abdulaziz University for Health Sciences, Ar Rimayah District, PO Box 22490, Riyadh 11426, Saudi Arabia; College of Medicine, King Saud bin Abdulaziz University for Health Sciences (KSAU-HS), Sheikh Jaber Al-Sabah Road, Khashm Al An District, PO Box 3660, Riyadh 11481, Saudi Arabia; College of Medicine, King Saud bin Abdulaziz University for Health Sciences (KSAU-HS), Sheikh Jaber Al-Sabah Road, Khashm Al An District, PO Box 3660, Riyadh 11481, Saudi Arabia; Division of Urology, Department of Surgery, King Abdulaziz Medical City, Ministry of National Guard Health Affairs, King Saud Bin Abdulaziz University for Health Sciences, Ar Rimayah District, PO Box 22490, Riyadh 11426, Saudi Arabia; King Abdullah International Medical Research Centre (KAIMRC), Ministry of National Guard Health Affairs, King Saud Bin Abdulaziz University for Health Sciences, Ar Rimayah District, PO Box 22490, Riyadh 11426, Saudi Arabia

**Keywords:** Zinner syndrome, seminal vesicle cyst, renal agenesis, robotic surgery, congenital anomaly

## Abstract

Zinner syndrome typically presents in young adulthood with nonspecific symptoms, making diagnosis challenging. A 21-year-old male presented with pelvic and lower abdominal pain. Imaging revealed a right seminal vesicle cyst measuring 8.2 × 6.4 × 5.1 cm and ipsilateral renal agenesis. The patient underwent robotic excision of the right seminal vesicle cyst with complete excision of a cyst containing 150 cc of fluid without complications, preserving adjacent anatomical structures. The precision and minimal invasiveness of this approach lead to optimal outcomes in treating this condition. Early recognition and surgical intervention are crucial for symptom resolution and improved quality of life.

## Introduction

Zinner syndrome is a rare congenital developmental anomaly resulting from malformation of the mesonephric (Wolffian) duct during embryological development. First described by Austrian urologist A. Zinner in 1914, it is characterized by the classic triad of unilateral seminal vesicle cyst, ipsilateral renal agenesis, and ejaculatory duct obstruction [1]. The condition arises from an insult occurring between the 4th and 13th gestational weeks, affecting the embryological development of both the reproductive and urinary systems due to their common embryological origin from the Wolffian duct.

While the exact prevalence remains unknown, fewer than 200 cases have been reported in the medical literature, making it an uncommonly encountered urological condition [2]. The syndrome typically manifests in the third or fourth decade of life when sexual activity increases and reproductive function becomes more relevant. Patients may present with a spectrum of symptoms, including pelvic pain, perineal discomfort, dysuria, painful ejaculation, hematospermia, or infertility. Some cases remain asymptomatic and are discovered incidentally during imaging studies for unrelated conditions.

Here, we present a case of Zinner syndrome in a young male who initially presented with chronic pelvic pain. Through this case, we discuss the diagnostic approach, imaging findings, and therapeutic considerations, highlighting the importance of maintaining a high clinical suspicion for this rare condition in young men presenting with unexplained urogenital symptoms, particularly when accompanied by renal anomalies.

## Case presentation

A 21-year-old male presented to the urology clinic with a 6-month history of progressive pelvic and lower abdominal pain. The pain was described as dull and constant, rated 6/10 in intensity, and exacerbated by prolonged sitting and sexual activity. His medical history was significant for an appendectomy nine years prior, with no other relevant surgical or medical conditions. The patient denied any urinary symptoms, hematuria, or hematospermia. On physical examination, he had normal external genitalia with bilaterally descended testes of normal size and consistency. The vas deferens were palpable bilaterally, and digital rectal examination revealed a soft, nontender prostate with fullness in the right seminal vesicle region.

## Preoperative investigations

Complete blood count, basic metabolic panel, and urinalysis were within normal limits. Semen analysis revealed sperm morphology of 2% normal forms (reference range: >4%), semen IgG of 10 (within normal range), and decreased semen viscosity of 2%. The semen volume was 1.5 ml (reference range: 1.5–5.0 ml).

**Figure 1:**
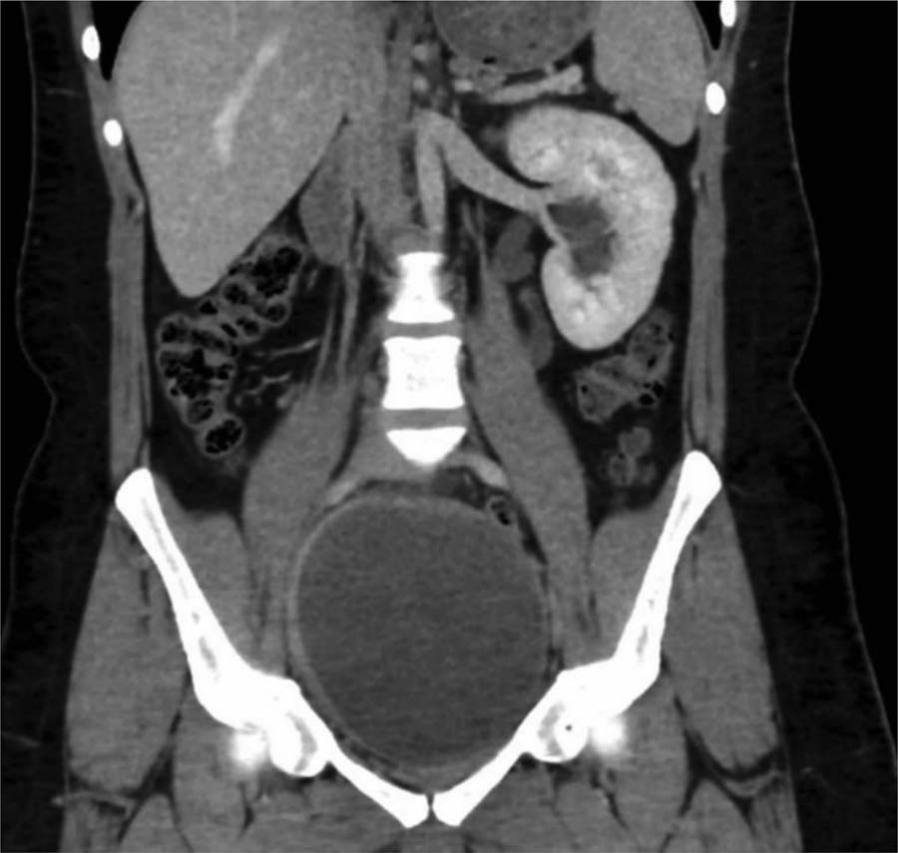
Coronal CT scan showing an enlarged right seminal vesicle cyst measuring 8.2 × 6.4 × 5.1 cm and ipsilateral renal agenesis.

Computed tomography (CT) of the pelvis demonstrated a large right seminal vesicle cyst measuring 8.2 × 6.4 × 5.1 cm, left-sided moderate hydroureteronephrosis, and absence of right renal or ureteral structures (renal agenesis). The left kidney has compensatory hypertrophy, and no other significant abdominal or pelvic abnormalities were noted (Fig. 1).

## Surgical management

**Figure 2:**
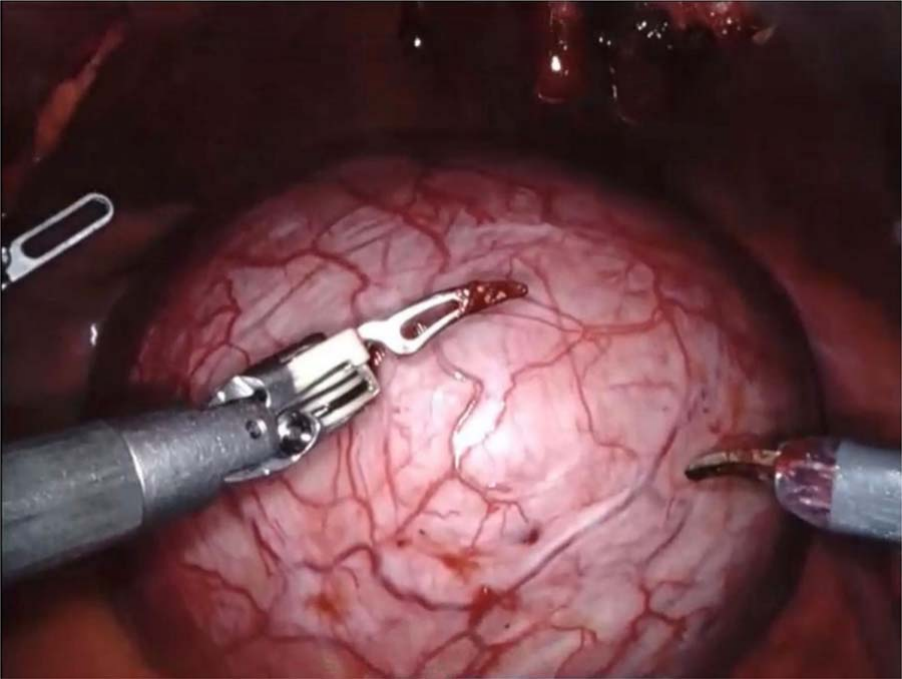
An intraoperative image showing a large right seminal vesicle cyst identified posterior to the bladder.

**Figure 3:**
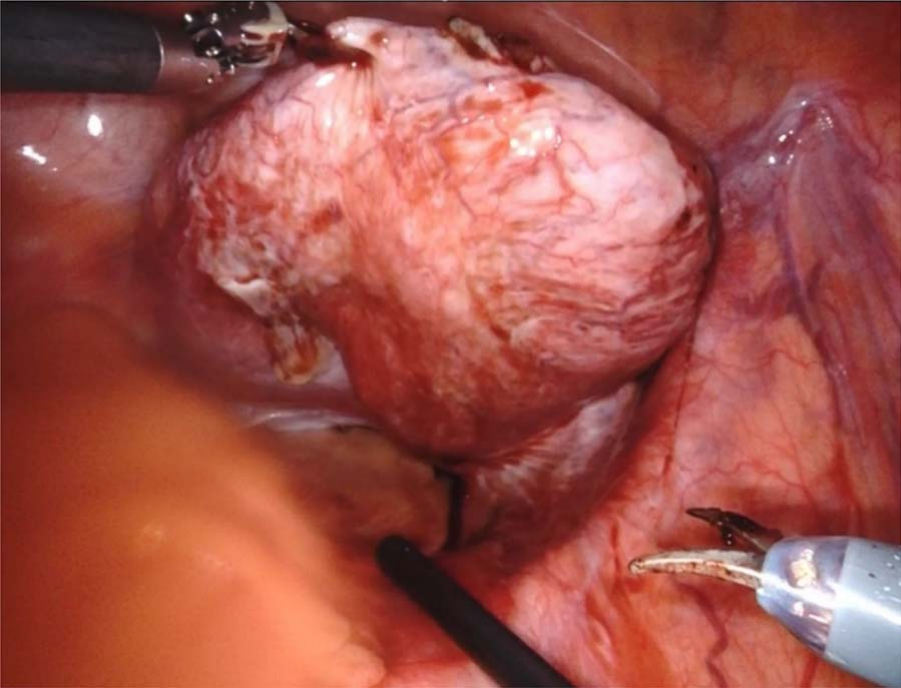
The seminal vesicle cyst after dissection of surrounding tissue and fluid aspiration (150 cc).

Based on the patient’s symptoms and imaging findings, robotic-assisted laparoscopic excision of the right seminal vesicle cyst was planned. The procedure was performed under general anesthesia using a da Vinci surgical system with a four-port approach. The patient was positioned in steep Trendelenburg position. A large right seminal vesicle cyst was identified posterior to the bladder (Fig. 2). Meticulous dissection was performed to separate the cyst from surrounding structures. The cyst contents were aspirated, yielding 150 cc of brownish fluid (Fig. 3). Complete excision was achieved while preserving the bladder neck, left ureter, and neurovascular bundles. Hem-o-lok clips were applied to secure the base of the cyst. Complete hemostasis was achieved, and there were no intraoperative complications.

## Discussion

Zinner syndrome is often underdiagnosed due to its rarity and nonspecific clinical presentation. The syndrome arises from a developmental insult occurring between the 4th and 13th gestational weeks, affecting the mesonephric duct, which gives rise to both the upper urinary tract and male reproductive organs. This embryological malformation results in the characteristic triad of ipsilateral renal agenesis, seminal vesicle cysts, and ejaculatory duct obstruction. The associated symptoms typically manifest in early adulthood and vary depending on the size and location of the seminal vesicle cyst, commonly including pelvic pain, urinary symptoms, ejaculatory dysfunction, or infertility.

The diagnostic approach to Zinner syndrome requires a high index of clinical suspicion and appropriate imaging studies. While ultrasound may initially detect renal absence or pelvic cysts, magnetic resonance imaging (MRI) has emerged as the gold-standard diagnostic tool. MRI provides superior soft tissue resolution, allowing detailed characterization of seminal vesicle cysts and their relationship to surrounding pelvic structures. In our case, however, CT confirmed the seminal vesicle cyst and the ipsilateral renal agenesis, establishing the diagnosis of Zinner syndrome.

The management of Zinner syndrome depends on the severity of symptoms and the size of the seminal vesicle cyst. Asymptomatic patients with small cysts may be managed conservatively with regular monitoring. However, surgical intervention is indicated for symptomatic cases, particularly those presenting with significant pain, infertility, or urinary symptoms. Traditional open surgical approaches have largely been superseded by minimally invasive techniques, with robotic-assisted laparoscopic surgery emerging as the preferred approach for managing symptomatic seminal vesicle cysts [3, 4].

The advantages of robotic-assisted surgery in treating Zinner syndrome are numerous. The enhanced three-dimensional visualization and precise instrument control allow for meticulous dissection in the deep pelvis, minimizing the risk of injury to adjacent structures such as the bladder, rectum, and neurovascular bundles. Additionally, the robotic platform enables better access to the seminal vesicles, which are anatomically challenging to reach through conventional approaches. This translates to reduced postoperative pain, shorter hospital stays, and faster recovery compared to open surgery.

Our case demonstrated several important technical considerations in the surgical management of seminal vesicle cysts. Initial cyst decompression through aspiration facilitated easier dissection and improved visualization of surgical planes. The use of Hem-o-lok clips for securing the cyst base provided reliable hemostasis. Careful attention to preserving adjacent structures, particularly the contralateral seminal vesicle and vas deferens, is crucial for maintaining reproductive function.

Long-term follow-up of patients with Zinner syndrome is important, particularly regarding fertility outcomes. While surgical excision typically resolves immediate symptoms, the impact on fertility may vary depending on the presence of contralateral abnormalities and the degree of ejaculatory duct obstruction. Additionally, patients should be monitored for potential complications related to their solitary kidney.

This case underscores the importance of considering Zinner syndrome in young males presenting with pelvic pain and urogenital symptoms, particularly when associated with renal anomalies. Early recognition and appropriate surgical intervention can significantly improve quality of life and potentially preserve fertility. As surgical techniques continue to evolve, robotic-assisted approaches offer an optimal balance of surgical precision and minimal invasiveness in the management of this rare condition.

## Conclusion

Zinner syndrome represents a rare but clinically significant congenital anomaly that requires a high index of suspicion for diagnosis, particularly in young men presenting with pelvic pain and urogenital symptoms. This case highlights the effectiveness of robotic-assisted laparoscopic excision in managing symptomatic seminal vesicle cysts, demonstrating excellent outcomes with minimal morbidity. The role of comprehensive imaging, particularly MRI, is crucial for accurate diagnosis and surgical planning. As awareness of this condition increases among clinicians, early recognition and appropriate surgical intervention can significantly improve patient outcomes and quality of life. Future research should focus on long-term follow-up studies to better understand the impact of surgical management on fertility outcomes and potential complications in this patient population.
